# Understanding and misunderstanding group mean centering: a commentary on Kelley et al.’s dangerous practice

**DOI:** 10.1007/s11135-017-0593-5

**Published:** 2017-11-07

**Authors:** Andrew Bell, Kelvyn Jones, Malcolm Fairbrother

**Affiliations:** 1 Sheffield Methods Institute, University of Sheffield, 219 Portobello, Sheffield, S1 4DP UK; 2 School of Geographical Sciences, University of Bristol, University Road, Bristol, BS8 1SS UK; 3 Department of Sociology, Umeå University, 901 87 Umeå, Sweden

**Keywords:** Multilevel models, Random effects, Group-mean-centering, Mundlak, Fixed effects

## Abstract

Kelley et al. argue that group-mean-centering covariates in multilevel models is dangerous, since—they claim—it generates results that are biased and misleading. We argue instead that what is dangerous is Kelley et al.’s unjustified assault on a simple statistical procedure that is enormously helpful, if not vital, in analyses of multilevel data. Kelley et al.’s arguments appear to be based on a faulty algebraic operation, and on a simplistic argument that parameter estimates from models with mean-centered covariates must be wrong merely because they are different than those from models with uncentered covariates. They also fail to explain why researchers should dispense with mean-centering when it is central to the estimation of fixed effects models—a common alternative approach to the analysis of clustered data, albeit one increasingly incorporated within a random effects framework. Group-mean-centering is, in short, no more dangerous than any other statistical procedure, and should remain a normal part of multilevel data analyses where it can be judiciously employed to good effect.

## Introduction

Kelley et al. (2017) argue that group-mean-centering variables in multilevel models is dangerous, concluding not just that centering should be used with caution but advocating “that group-mean centering be abandoned” (p. 281) entirely. Our commentary takes issue with this bold recommendation, and supports instead the opposite view: that group-mean-centering is substantially more informative than fitting models using “raw” (or, uncentered) variables, such that group-mean-centering should be standard practice.

We begin our commentary with a review of the basic reasons to group-mean-centre covariates in multilevel models, and of the substantive meaning of the coefficients on both the group-mean-centred variable, and the group-mean variable often included with it. We explain why it should come as no surprise that the estimated coefficients of these are different to those estimated on raw, uncentered variables, and we note that many analysts of clustered or multilevel data employ fixed effects models precisely because they do not trust uncentered covariates. Finally, we identify a flaw in Kelley et al.’s algebra, which may also help explain their erroneous conclusions and claims, including their suggestion that models with mean-centered covariates are constrained whereas those with uncentered covariates are unconstrained.

## Group mean centering and within, between and contextual effects

Table 1 shows a range of different models that can be used to analyse multilevel data. In each case, *i* represents level 1 entities (for example individuals) and *j* represents level 2 entities (for example countries). Covariate *x*_*ij*_ is a predictor measured at level 1, and *y*_*ij*_ is a response variable, also measured at level 1. *u*_*j*_ is the level 2 random effect, and *e*_*ij*_ is the level 1 random effect. Each model (except model 5) includes an intercept *β*_0_, and one (or more) measure of the effect of *x*_*ij*_ on *y*_*ij*_.^1^

**Table 1:** Comparison of different models

Model name	Model
1. Standard RE	$y_{ij} = \beta_{0} + \beta_{R} x_{ij} + \left( {u_{j} + e_{ij} } \right)$
2. Within	$y_{ij} = \beta_{0} + \beta_{W} \left( {x_{ij} - \bar{x}_{j} } \right) + \left( {u_{j} + e_{ij} } \right)$
3. Mundlak	$y_{ij} = \beta_{0} + \beta_{W} x_{ij} + \beta_{C} \bar{x}_{j} + \left( {u_{j} + e_{ij} } \right)$
4. Within–between	$y_{ij} = \beta_{0} + \beta_{W} \left( {x_{ij} - \bar{x}_{j} } \right) + \beta_{B} \bar{x}_{j} + \left( {u_{j} + e_{ij} } \right)$
5. Fixed effects	$\left( {y_{ij} - \bar{y}_{j} } \right) = \beta_{W} \left( {x_{ij} - \bar{x}_{j} } \right) + \left( {e_{ij} } \right)$

Kelley et al. argue in favour of the standard RE model (model 1), over the model with group-mean-centering, model 2. The limitation of their preferred model, however, is that the estimated coefficient on *x*_*ij*_ is a weighted average of two effects—one at level 1 and the other at level 2 (Bell et al. 2016; Bell and Jones 2015; Fairbrother 2014). These two effects have different substantive meanings; they capture the within-group and between-group relationships, respectively, and these relationships may be quite different. At level 1, the ‘within’ effect captures the difference on Y between units that are higher or lower *than average on X relative to their group*, whilst at level 2 the ‘between’ or ‘contextual’^2^ effect captures the difference between *groups that have a higher or lower X as a whole (or, equivalently, on average)*. In for example Kelley et al.’s example 5, the within effect is the effect of being more religious than the country average, compared to the between effect of living in a more religious country. There is no reason to expect these two associations to be the same, either because of different substantive processes happening at each level, or because of unmeasured confounding variables affecting the estimate at the higher or lower level. For example, consider pupil self-worth, which can be modelled as a function of prior attainment at both the individual (within) and school class (between) levels. The findings are unequivocal: the big-fish–little-pond effect correctly predicts that equally able students have lower academic self-concept in schools where the average ability level of classmates is high, but higher where the school-average ability is lower (Marsh et al. 2008; Seaton et al. 2010). One effect is not reducible to the other: the within effect (level 1) is positive, whilst the contextual (level 2) effect is negative.

Whilst Kelley et al. acknowledge the difference between the within and between effects in places, in the main they disregard it and instead claim that the relationship of true interest is the simple effect of (raw) X on Y (e.g., the effect of being more religious). What they do not acknowledge, however, is that there are *always* two distinct relationships. To find that the coefficients estimated using centered and uncentered models differ is merely proof that the within effect is not the same as a weighted average of the within and between effects—meaning, by implication, that the within effect is also not the same as the between effect. The within effect revealed by group-mean-centering a covariate is not therefore wrong; it is answering a different question, capturing a different relationship than the weighted average of the between and within effects reflected in the coefficient on the uncentered covariate. Kelley et al. do not realise that attempting to combine these two relationships inevitably leads to biased estimates of both of them, since each will be drawn towards the other. This is the fundamental reason why we, and others (Allison 2009; Bartels 2015; Bell et al. 2016; Bell and Jones 2015; Enders and Tofighi 2007), have argued in favour of group mean centering, and this view is supported by simulation studies: it is a means of isolating and differentiating distinct within and between relationships.

Many researchers attach such importance to the isolation of the within relationship when analysing clustered data that they use models designed specifically for it: fixed effects (FE) models (the fifth type in Table 1). Such models are regarded by some as the ‘gold standard’ method for observational research (e.g., Schurer and Yong 2012, p. 1), arguably because it is safer to interpret within rather than between effects as causal. While, formally, FE models include a series of dummy variables for the groups, in practice estimation is usually based precisely on group-mean-centering as shown in Table 1. From this perspective it is nothing short of bizarre for Kelley et al. to vilify group-mean-centering, the application of which in multilevel (also known as random effects) models is merely the strategic appropriation of the elegantly simple but brilliant idea at the heart of fixed effects models—models that have been widely employed across the social sciences for many years.

Employing group-mean-centering in a random effects rather than fixed effects framework, moreover, allows for more flexibility in the investigation of other relationships. Above all, multilevel models can additionally include the group mean of a lower-level covariate to test for a ‘between’ relationship—as in model 4 in Table 1 above.

Kelley et al. present a number of examples, in each case showing two sets of (differing) results, which they then take as proof that models using raw/uncentered X are correct and those using group-mean-centering are incorrect. In places they point out that models using the raw X have a higher R-squared value and/or a lower Variance Partitioning Coefficient (VPC). However neither of these findings are surprising: they simply reflect the nature of the variables, not the correctness of one or the other estimate. A group-mean-centered variable has no variance at the group level (by definition), meaning that it will explain no variance in the outcome at that level. It is therefore unsurprising that models with group-mean-centered covariates (and no group-mean covariate) explain less variance, both generally (R-squared) and at the higher level specifically (VPC). Introducing the group mean as a higher level variable with a ‘between’ effect would increase the R-squared, and reduce the VPC. Kelley et al. also seem surprised that “group-mean centering gives correlations near zero” between group-mean-centered variables and other higher-level variables. But, again, group-mean centered variables have no variance at the higher level—so how could they correlate with anything at that level?

Mathematically, Kelley et al.’s entire argument rests on the claim that a model with a mean-centered *x* is a constrained model, a claim they purport to justify theoretically on pp. 278–279. Here they present a series of equations on the basis of which they suggest that models with a group-mean centered *x* “will give an unbiased estimates [sic] only if the constraint $b_{{1^{\prime } }} { = } - b_{{2^{*} }}$ holds”—where by $b_{{1^{\prime } }}$ we assume them to mean $b_{{1^{*} }}$ in their Equation 6b. They therefore believe that a model with a mean-centered *x* yields unbiased and meaningful estimates only if the data-generating process possesses the highly unlikely property of incorporating two coefficients equal in magnitude but opposite in sign.

Kelley et al. have it exactly backwards: it is the uncentered model that is constrained, whereas the mean-centered model is unconstrained. The mean-centered specification neither imposes nor assumes any relationship between the coefficients on the two components of *x*; on the contrary, it specifically permits the coefficients on them to differ. In contrast, not mean-centering *x* imposes the constraint that the coefficients on the two components must be identical. This can easily be seen by comparing the two models (using Kelley et al.’s preferred notation):

$$y = b_{0} + b_{1} IndividualX + b_{3} CountryZ + Error$$

and

$$y = b_{{0^{*} }} + b_{{1^{*} }} IndividualXCentered + b_{{2^{*} }} CountryMeanX + b_{{3^{*} }} CountryZ + Error.$$

The second (mean-centered) model includes one more parameter than the uncentered model, and does not require b1* to equal b2*, demonstrating its greater flexibility.

Kelley et al.’s misunderstanding about the relative flexibility of the two specifications may be due to an error of algebra. They suggest that their equation 5 derives from the substitution of their equation (4) into their equation (3). But it does not. Substituting (4) into (3) in fact yields:

$$y = b_{0} + b_{1} \left( {IndividualXCentered + CountryMeanX} \right) + b_{3} CountryZ + Error$$

which can be rearranged to

$$y = b_{0} + b_{1} IndividualXCentered + b_{1} CountryMeanX + b_{3} CountryZ + Error.$$

This again shows the constraint imposed by the uncentered model—the coefficients on the two components of *x* are constrained to be the same—thereby demonstrating the undesirable properties of the standard RE model, Kelley et al's equation 3. Instead of this constrained model, what we in fact want is to substitute (4) into (3), and then estimate separate coefficients on *IndividualXCentered* and *CountryMeanX*—that is, *b*1* and *b*2* above, rather than *b*1 alone.

Kelley et al’s equation (5) does not reflect the substitution of (4) into (3), but it is (as Kelley et al. say) a model of *y* as a function of a mean-centered *X*. Kelley et al. argue that this is a special case of the more flexible (unconstrained) model 6b—which, as we recommend, includes both *b*1 and *b*2. Intriguingly, in 6b, Kelley et al. appear to have hit on a specification first derived in 1978 (by Mundlak)—that is mathematically equivalent to a mean-centering-based solution to the well-known problem of endogeneity bias (see Bell and Jones 2015, pp. 141, 142). Kelley et al. say there is precisely one circumstance in which the estimate on the coefficient $b_{{1^{\prime } }}$ in Equation 5 is equal to $b_{{1^{*} }}$ in Equation 6b: where $b_{{1^{*} }}$ is estimated to be equal to $- \;b_{{2^{*} }}$. That, however, is incorrect; rather, the coefficient $b_{{1^{\prime } }}$ in Equation 5 is always equal to $b_{{1^{*} }}$. The reason is that, by including *CountryMeanX*, 6b controls for the group-mean component of *IndividualX*—just like the inclusion of *CountryMeanX* does in 5. This can be verified using any dataset, whether real or simulated. In fact, even if *IndividualXCentered* were to be substituted for *IndividualX*, the estimate for $b_{{1^{*} }}$ would be the same (though the estimate for $b_{{2^{*} }}$ would change). This is because, in either case, the within-individual variation is removed from the variable, either by controlling for *CountryMeanX* (as in Kelley et al’s equation 6b), or by group-mean-centering the variable *IndividualX* (equation 5), or both.

The ‘Mundlak’ specification (Mundlak 1978, model 3 in Table 1) gives the within effect and the ‘contextual’ effect (the latter being the higher level effect controlling for the raw value of an individual; see Bell et al. 2016, p. 8, for more on the difference between contextual and between effects). Both the higher- and lower-level effects can be of substantive interest (as illuminated by either model 3 or 4 in Table 1). But whilst Kelley et al. commend the Mundlak model at various points, they fail to acknowledge that it (a) produces results different from the standard multilevel model (model 1 in Table 1), (b) produces identical within effects to the within model (model 2) to which they are opposed, and (c) is mathematically equivalent to the within–between model that uses group-mean-centering. They also fail to acknowledge the importance of including the group-mean variable in the model to achieve unbiased within effects.

The only thing that weighs against what we have said here is Kelley et al.’s table 6. Here they present a Mundlak-type model with the group mean included as a higher-level variable. They show that the results match those for the raw-X model, and not the group-mean-centered-X model. We do not know how they found this result: it contradicts all simulations and real-data models that we have run and published. As such, we presume that there is some typographical error at work here.^3^

## Summary

We have shown in this comment why Kelley et al. are wrong to suggest that group-mean-centering should be abandoned. Models with group-mean centering are not in any sense incorrect or misleading; rather, they investigate different (more) relationships than models using raw, uncentered covariates, yielding richer and more reliable insights about the relationships present in multilevel datasets. It is important to understand the “multilevel” in multilevel models: that is, covariates at different levels can be related to outcome variables in quite different ways, and mean-centering them allows and tests the relationships at a specific level. Researchers are now making routine use of group-mean-centering—usually including the group-mean of lower-level variables as an additional higher level variable to illuminate the ‘between’ effect—and they should continue to do; contrary to Kelley et al. it would be dangerous to stop. What Kelley at el are advocating is confounding two sources of variation into one and treating the results as homogenous—the exact opposite of what is needed to appreciate the nature of multi-level effects.

## Footnotes

1 We should clarify that we use the term “effect” here in a loose sense, not necessarily implying a causal relationship.

2 The difference between the between (*β*_*B*_) and contextual (*β*_*C*_) effect are discussed further later; see also Bell et al. (2016, p. 8). In general, we are interested in one or the other, depending on the research question and data structure.

3 Whilst we contacted the authors for clarification on this, no reply was forthcoming.
